# Supplementary material for: Path analysis model for preventing stunting in dryland area island East Nusa Tenggara Province, Indonesia
Source: PLoS One. 2023 Nov 2;18(11):e0293797. doi: 10.1371/journal.pone.0293797 (PMC10621912; doi:10.1371/journal.pone.0293797)
Supplement: Attachment — Submitted filename: Authors response to Reviewers v1.docx [file pone.0293797.s001.docx]

**Dear Reviewers,**

Thank you for giving us the opportunity to improve and resubmit our manuscript “*Path Analysis Model for Preventing Stunting in Dryland Area Island East Nusa Tenggara Province*”. Please find enclosed the revised manuscript for further consideration. The manuscript has been revised according to the comments raised by the reviewer. We would like to thank the reviewer for the constructive and competent criticism, and we hope that our manuscript will be acceptable for publication in PLOS ONE.

Reviewers' comments:

Reviewer's Responses to Questions

Comments to the Author

1. Is the manuscript technically sound, and do the data support the conclusions?

The manuscript must describe a technically sound piece of scientific research with data that supports the conclusions. Experiments must have been conducted rigorously, with appropriate controls, replication, and sample sizes. The conclusions must be drawn appropriately based on the data presented.

Reviewer #1: Yes

**Response:** Thank you for your comments regarding our manuscript.

2. Has the statistical analysis been performed appropriately and rigorously?

Reviewer #1: I Don't Know

**Response:** Thank you for providing these insights. We have now the statistical analysis was revised and has been changed. We think these changes now better. We hope that you agree.

3. Have the authors made all data underlying the findings in their manuscript fully available?

The PLOS Data policy requires authors to make all data underlying the findings described in their manuscript fully available without restriction, with rare exception (please refer to the Data Availability Statement in the manuscript PDF file). The data should be provided as part of the manuscript or its supporting information, or deposited to a public repository. For example, in addition to summary statistics, the data points behind means, medians and variance measures should be available. If there are restrictions on publicly sharing data—e.g. participant privacy or use of data from a third party—those must be specified.

Reviewer #1: No

**Response:** Thank you for your suggestion. We will provide the information about the data as part of the manuscript or its supporting information and will put the information regarding our publicly sharing data statement since this data came from a third party.

4. Is the manuscript presented in an intelligible fashion and written in standard English?

PLOS ONE does not copyedit accepted manuscripts, so the language in submitted articles must be clear, correct, and unambiguous. Any typographical or grammatical errors should be corrected at revision, so please note any specific errors here.

Reviewer #1: Yes

**Response:** Thank you for your comments regarding our manuscript.

**5. Review Comments to the Author**

Please use the space provided to explain your answers to the questions above. You may also include additional comments for the author, including concerns about dual publication, research ethics, or publication ethics. (Please upload your review as an attachment if it exceeds 20,000 characters)

Reviewer #1: The paper is very interesting in which the authors have presented a stunting prevention model for a dry area of East Nusa Tenggara Province, Indonesia. My further comments are as below:

(i) There are several grammatical/ typographical errors and it need a proof read. For example, Figure 1, Nutrition specific intervention programe..... "chek up", it should be check-up. Line 142, model formulation ----in this study?. Line 182, with thereby ndicator...

**Response:** Thank you for your suggestion. We have amended grammatical/ typographical errors in our manuscript.

(ii) why you have recommended the value of loading factor as 0.6. What is the significance of this value?

**Response:**

The standardized loading factor show how well an item represents the underlying construct. The standardized loading factor describes the magnitude of the correlation between each measurement item (indicator) and its construct. The loading factor value > 0.7 is said to be ideal, meaning that the indicator is said to be valid in measuring the construct. In empirical research, the loading factor value > 0.5 is still acceptable. Thus, the loading factor value < 0.5 must be removed from the model (dropped).

(iii) In Table 3, Micro> Food access has P-value=0.087>0.05, but you have mentioned in line 223-224 that Food access variable have significant to nutritional intake. Can you explain this?

**Response:**

We stated in our manuscript that the macro determinant variables to be insignificant factors to food access. Moreover, in our analysis found that food access have significant to nutritional intake. Food access plays a crucial role in determining an individual's nutritional intake. It refers to the ability of people to obtain and utilize sufficient, safe, and nutritious food to meet their dietary needs. Several factors can influence food access, including geographic location, income level, transportation, and availability of food retailers. Improving food access is vital for promoting healthier diets and reducing the risk of malnutrition and diet-related diseases. Efforts to address food access disparities include increasing the availability of affordable, nutritious foods in underserved areas, promoting nutrition education, supporting local food systems, and implementing policies that encourage equitable food access for all individuals.

(iv) Is it not helpful, among others, to increase health care units/hospitals to cope with stunting in the province? As parallel to food security there is also need of establishing health care units.

**Response:**

We agree with you that increasing health care units/hospitals to cope with stunting in the province can be helpful in addressing stunting in a province. Stunting is a complex issue that arises from multiple factors, including inadequate nutrition, poor healthcare access, and limited sanitation and hygiene facilities. To effectively combat stunting, a comprehensive approach is required, which includes both improving food security and ensuring access to quality healthcare. By establishing additional healthcare units and hospitals, we can enhance the availability and accessibility of healthcare services for the population in the province. These facilities can provide essential medical care, including prenatal and postnatal care, immunizations, nutritional counselling, and treatment for illnesses and infections that contribute to stunting. They can also support early identification and intervention for children who are at risk of or experiencing stunted growth.

Moreover, healthcare units and hospitals play a crucial role in promoting health education and awareness programs. They can educate families and caregivers about proper nutrition, hygiene practices, and the importance of breastfeeding, among other key factors that contribute to child growth and development. By integrating healthcare services with nutrition interventions, we can effectively address stunting and improve the overall health and well-being of the population. However, it is important to note that simply increasing the number of healthcare units and hospitals is not sufficient on its own. Adequate staffing, training, and resources are also essential to ensure the effective functioning of these facilities. Collaboration with other sectors such as agriculture, education, and social welfare is also crucial for a comprehensive approach to combating stunting. In summary, establishing more healthcare units and hospitals can be an important component of addressing stunting in a province. However, it should be part of a broader strategy that includes improving food security, enhancing healthcare access, promoting health education, and fostering multisectoral collaboration to achieve sustainable results in reducing stunting rates.

6. PLOS authors have the option to publish the peer review history of their article (what does this mean?). If published, this will include your full peer review and any attached files.

If you choose “no”, your identity will remain anonymous but your review may still be made public.

Do you want your identity to be public for this peer review? For information about this choice, including consent withdrawal, please see our Privacy Policy.

Reviewer #1: No

[NOTE: If reviewer comments were submitted as an attachment file, they will be attached to this email and accessible via the submission site. Please log into your account, locate the manuscript record, and check for the action link "View Attachments". If this link does not appear, there are no attachment files.]

While revising your submission, please upload your figure files to the Preflight Analysis and Conversion Engine (PACE) digital diagnostic tool, https://pacev2.apexcovantage.com/. PACE helps ensure that figures meet PLOS requirements. To use PACE, you must first register as a user. Registration is free. Then, login and navigate to the UPLOAD tab, where you will find detailed instructions on how to use the tool. If you encounter any issues or have any questions when using PACE, please email PLOS at figures@plos.org. Please note that Supporting Information files do not need this step.
