# Supplementary material for: Path analysis model for preventing stunting in dryland area island East Nusa Tenggara Province, Indonesia
Source: PLoS One. 2023 Nov 2;18(11):e0293797. doi: 10.1371/journal.pone.0293797 (PMC10621912; doi:10.1371/journal.pone.0293797)
Supplement: Attachment — Submitted filename: Authors response to Reviewers v2.docx [file pone.0293797.s002.docx]

**Dear Reviewers,**

Thank you for giving us the opportunity to improve and resubmit our manuscript “*Path Analysis Model for Preventing Stunting in Dryland Area Island East Nusa Tenggara Province*”. Please find enclosed the revised manuscript for further consideration. The manuscript has been revised according to the comments raised by the reviewer. We would like to thank the reviewer for the constructive and competent criticism, and we hope that our manuscript will be acceptable for publication in PLOS ONE.

Reviewers' comments:

Reviewer's Responses to Questions

Comments to the Author

1. If the authors have adequately addressed your comments raised in a previous round of review and you feel that this manuscript is now acceptable for publication, you may indicate that here to bypass the “Comments to the Author” section, enter your conflict of interest statement in the “Confidential to Editor” section, and submit your "Accept" recommendation.

Reviewer #1: (No Response)

2. Is the manuscript technically sound, and do the data support the conclusions?

The manuscript must describe a technically sound piece of scientific research with data that supports the conclusions. Experiments must have been conducted rigorously, with appropriate controls, replication, and sample sizes. The conclusions must be drawn appropriately based on the data presented.

Reviewer #1: Yes

**Response:** Thank you for your comments regarding our manuscript.

3. Has the statistical analysis been performed appropriately and rigorously?

Reviewer #1: Yes

**Response:** Thank you for your valuable insight regarding our manuscript.

4. Have the authors made all data underlying the findings in their manuscript fully available?

The PLOS Data policy requires authors to make all data underlying the findings described in their manuscript fully available without restriction, with rare exception (please refer to the Data Availability Statement in the manuscript PDF file). The data should be provided as part of the manuscript or its supporting information, or deposited to a public repository. For example, in addition to summary statistics, the data points behind means, medians and variance measures should be available. If there are restrictions on publicly sharing data—e.g. participant privacy or use of data from a third party—those must be specified.

Reviewer #1: Yes

**Response:** Thank you for your valuable insight regarding our manuscript.

5. Is the manuscript presented in an intelligible fashion and written in standard English?

PLOS ONE does not copyedit accepted manuscripts, so the language in submitted articles must be clear, correct, and unambiguous. Any typographical or grammatical errors should be corrected at revision, so please note any specific errors here.

Reviewer #1: Yes

**Response:** Thank you for your comments regarding our manuscript.

6. Review Comments to the Author

Please use the space provided to explain your answers to the questions above. You may also include additional comments for the author, including concerns about dual publication, research ethics, or publication ethics. (Please upload your review as an attachment if it exceeds 20,000 characters)

Reviewer #1: The authors have not categorically address my final query ((iv)- Is it not helpful, among others, to increase health care units/hospitals to cope with stunting in the province? As parallel to food security there is also need of establishing health

care units). Anyhow, I suggest them to include their point of view, that is also quite reasonable, in an appropriate place of the paper to facilitate the reader(s).

**Response:** Thank you for your suggestion. We will provide the information about your final query as part of the manuscript. We agree with you that increasing health care units/hospitals to cope with stunting in the province can be helpful in addressing stunting in a province. We have now put your suggestion in the paper.

7. PLOS authors have the option to publish the peer review history of their article (what does this mean?). If published, this will include your full peer review and any attached files.

If you choose “no”, your identity will remain anonymous but your review may still be made public.

Do you want your identity to be public for this peer review? For information about this choice, including consent withdrawal, please see our Privacy Policy.

Reviewer #1: No
